# Revised methods guide for economic evaluation studies of health technologies in Portugal

**DOI:** 10.1017/S0266462325103413

**Published:** 2026-01-06

**Authors:** Marta O Soares, Julian Perelman, Ceu Mateus, Ana Duarte, Rita Faria, Lara N. Ferreira, Pedro Saramago, Paula Veiga Benesch, Claudia Furtado, Maria do Céu Teixeira, Sónia Caldeira, Mark Sculpher

**Affiliations:** 1 Centre for Health Economics, University of York, UK; 2 Escola Nacional de Saude Publica, Universidade Nova de Lisboa, Portugal; 3 Department of Health Research, Lancaster University, UK; 4 Univ Algarve ESGHT, Portugal School of Management, Hospitality and Tourism. University of Algarve, Portugal; 5 School of Economics and Management, University of Minho, Portugal; 6 Pricing and Reimbursement Division, Portuguese National Authority for Medicines and Health Products (INFARMED), Portugal

**Keywords:** economic evaluation, methods guide, Portugal, health technology assessment, INFARMED, decision making, health economics

## Abstract

**Introduction:**

Economic evaluation supports public funding decisions about the use of health technologies within the Portuguese National Health System (NHS). The methods guide for economic evaluation in Portugal serves both companies preparing economic evaluation submissions and the independent commission appraising the evidence submitted.

**Methods:**

This article presents the revised methods guide for economic evaluation in Portugal. The revisions reflect advances in economic evaluation, updates to regulatory policies, and responses to the evolving economic context. The paper highlights the most significant changes to the guidance, comparing the new Portuguese guidelines to those from the United Kingdom and Canada. The discussion is framed around key comments received during public consultation.

**Results:**

The updated guidelines recommend cost-effectiveness analyses based on quality-adjusted life years and advocate for long-term modelling, a 4 percent discount rate, and a focus on NHS costs. New features include guidance on the identification and management of uncertainty within a dynamic appraisal process with regular contract negotiations (which can trigger reappraisals). The guide also covers how cost-effectiveness models, typically centrally developed, should be adapted to the Portuguese context. It highlights the key role of structured expert elicitation to address uncertainties in evidence, including those related to model adaptation.

**Conclusions:**

The revision was developed through stakeholder consultations and aligns with international best practices, offering more explicit and transparent methods to support health resource allocation decisions.

## Introduction

In Portugal, economic evaluation has been used since the 1990s as part of a formal Health Technology Assessment (HTA) process. This process supports decisions on the financing of pharmaceuticals by the Portuguese National Health System (NHS), a tax-funded, universal healthcare system, that provides universal care that is (mostly) free at the point of use.

The appraisal process is currently undertaken by the Health Technology Assessment Commission (CATS), which is part of INFARMED (Autoridade Nacional do Medicamento e Productos de Saude, I.P.), a public regulatory agency. It is comprised of two evaluation steps, with the company submitting evidence for each. The first step aims to determine whether the drug provides added therapeutic value in relation to the current standard of care. Drugs that demonstrate added therapeutic value move on to the second step where they are assessed, using economic evaluation, to determine whether public funding of the drug of value to the health system. Independent expert reviewers (doctors, pharmacists, and economists) prepare clinical and cost-effectiveness appraisal reports. The reports are used by decision-makers, the INFARMED Board of Directors and, subsequently, the Portuguese Ministry of Health, to support price negotiations with the company. The negotiations establish a total expenditure cap (a function of price and of the size of the eligible population) for the duration of the contract (typically of 2 years), and any evidence requirements for reappraisal. While the evidence requirements for the evidence submitted are made explicit in the methods guide, the basis, and grounds for the decisions taken are not explicit. For example, cost-effectiveness threshold(s) are not publicly known (1).

The first Portuguese economic evaluation methods guide – one of the first in Europe – was published in 1998 (2). It supported the development and appraisal of economic evaluation studies for over 20 years. In this article, we report on the revision of the methods guide, which was warranted for a variety of reasons. First, recent regulatory policies enable certain technologies to be marketed earlier in the evidence development pathway when there is still significant uncertainty over their clinical and cost-effectiveness. This means that it is critical to create incentives for postfinancing evidence collection and to define clear principles for reappraisal. Second, the methods guide needed to better reflect advances in the theory and practice of economic evaluation, such as new techniques to handle uncertainty, model long-term effects, synthesize evidence, and more accurately measure treatment effects. Third, the Portuguese context has also changed over time. Multiple budgetary cuts imposed over recent years motivated the need to include budget impact analyses (BIAs) and to set up a formal reappraisal process. Finally, with an increase in the use of centrally developed cost-effectiveness models, there is a need to more explicitly consider sources of evidence specific to the Portuguese context and to harmonize the methods for eliciting expert judgements according to best practice.

This article presents the revised Portuguese methods guide for economic evaluation (published in full in (3)), which supports companies in developing their economic evaluation’ submissions and guide the appraisal process of new pharmaceuticals by the HTA commission (CATS). We here focus on the more notable changes and will only briefly touch upon aspects of the guidance that are largely unchanged. Throughout, we will compare the new Portuguese guidelines with those from the United Kingdom and Canada, which also present detailed guidance. Note that choices made in the development of the Portuguese methods guide recognized that the political, economic, and healthcare contexts differ significantly between these countries. Portugal is a smaller country, with lower GDP per capita, lower health expenditure, and a lower public investment in health. This should guide interpretation of differences between countries. The discussion is structured around the main comments received from stakeholders at public consultation.

## How was the methods guide developed?

In 2018, a working group of all of CATS’ expert economic reviewers, alongside an expert in health economics external to CATS (Mark Sculpher), developed a proposal for the revised methods guide for INFARMED and key public stakeholders to consider.

The process used was as follows. The working group started by identifying the topics to be considered. Subgroups of experts were then allocated to topics and were asked to: (1) produce a short overview on the relevance of the topic, (2) produce a summary of the 1998 guidelines and guidelines from other countries, (3) conduct a (nonsystematic) literature review on recent developments on the topic, and (4) identify possible options for guidance. A 2-day meeting was held in April 2018, where each group briefly presented their results, motivating discussion with a view to reach consensus on how to revise the guidance. The working group drafted the first version of the revised methods guide, which was reviewed by the leads of the clinical appraisal step of the process (to ensure consistency between clinical and cost-effectiveness guidelines) in October 2018. The resulting document was considered by INFARMED, I.P.’s Board of Directors, the Assistant Secretary of State for Health (at the time, Dr Francisco Ramos), and underwent further revision following consultation with a panel of selected key stakeholders (e.g., representatives of the pharmaceutical industry, academia, patient’ associations, other Portuguese Ministries, and public entities). These stakeholders were invited to submit written comments between May and June 2019 and present them in a public workshop held at INFARMED, I.P., in July 2019. The document was revised accordingly, to produce the published version (3).

## The updated methods guide to economic evaluation

As for many other guidelines across Europe and beyond (Table 1), the new Portuguese methods guide recommends cost-effectiveness analyses with health consequences expressed as quality-adjusted life years (QALYs). It recommends the use of a lifetime time horizon so that the impact of health technologies over the long-term is accounted for, and an annual discount rate of 4percent for both costs and consequences. The guidance acknowledges that the appraisal process aims to provide support for resource allocation decisions within the Portuguese NHS and, therefore, sets out a reference case where costs considered are those that fall on the NHS (including long-term care or palliative care costs, also financed by the NHS). However, costs unrelated to the disease, such as future unrelated costs associated with remaining alive, are not included in the reference case. Health consequences, or costs, for other or future patients (e.g., those infected by current patients), families and informal caregivers are also excluded from the reference case. The guidance allows for the possibility of restricting reimbursement to subgroups of patients when there is evidence of heterogeneity (in treatment effects and/or on other model parameters).

**Table 1.** Cross-country comparison of economic evaluation guidelines for key parameters

	Portugal 1999 (2)	Portugal 2019 (3)	UK 2022 (4)	Canada 2017 (5)
Effectiveness measure	QALYs preferred, followed by monetary values (cost–benefit analysis)	QALYs	QALYs	QALYs are recommended, others allowed including monetary values (cost–benefit analysis)
Comparators	The most common, most effective, and cheapest therapy	All therapies in current practice, including off-label and nonfinanced	All therapies in current practice, including off-label and nonfinanced	All therapies in current practice, if financed and commonly used
Time horizon	Sufficiently long to capture all costs and outcomes	Lifetime	Sufficiently long to capture differences between the technologies (lifetime recommended)	Sufficiently long to capture differences between the technologies (lifetime recommended)
Discounting for costs and consequences	5%	4%	3.5% (nonreference case with 1.5%)	1.5% (recommended)
Perspective	Societal preferred	NHS. Costs and benefits accrued by other stakeholders may be presented, but not included in ICER calculations	NHS and Personal Social Services (PSS), nonreference cases may include other government bodies’ and societal perspectives (e.g., health effects on carers)	Public payer for the reference case, other perspectives allowed for nonreference cases
Source of clinical evidence	RCTs preferred, nonrandomized studies allowed (unspecified cases)	RCTs preferred, nonrandomized studies allowed in specific cases. Systematic reviews required to identify sources of evidence	RCTs preferred, nonrandomized studies allowed in specific cases. Systematic reviews required to identify sources of evidence	Various sources allowed, including RCTs, observational studies, administrative databases, noncomparative studies, or expert input
QoL measure and valuation	No preferred measure	EQ–5D–5L with Portuguese tariffs preferred	EQ–5D–3L preferred	Utilities based on any generic classification system in reference case
Costs unrelated to the disease	Not considered for ICER calculation	As additional information, not included in ICER calculations	Not included.	Allowed in nonreference cases
Costs for families, caregivers, future generations	May be considered for the ICER calculation	As additional information, not included in ICER calculations	Allowed in nonreference cases (see Perspective)	Allowed in nonreference cases
Uncertainty	DSA	DSA, scenarios, PSA, EVPI, EVPPI	Scenarios, PSA (DSA if linear model), threshold analysis	PSA, scenarios, EVPI
Reappraisal	No guidance	Identification of additional evidence to be collected for reappraisal	Planned future evidence generation for uncertain parameters	Value of Information support definition of further research requirements for future decisions
Evidence to support decision-making	No guidance	ICER, threshold analysis on price, and price at which EVPI nonsignificant	ICER, net health benefits	No guidance
Budget impact analysis	No guidance	Guidance	Guidance	No guidance
Equity	No guidance	No guidance	Equal weights in reference case, but differential weights allowed in specific circumstances	Equal weights in reference case, but subgroup analyses with full description of subpopulations, to inform equity-related deliberation

In the following sections, we highlight novel aspects of this new guidance.

### Evaluation principles

The new Portuguese methods guide aims to ensure that the information in the submission optimally supports the decisions required. It sets out the appraisal process as dynamic, informing not only a first appraisal but also any possible contract renegotiations (reappraisals). To support such a dynamic process, besides determining cost-effectiveness to support reimbursement decisions for the duration of the contract, the new methods guide adds the key objective of determining what are relevant, appropriate, and feasible evidence development activities to support the next appraisal point. This informs formal requests for further evidence collection. To this effect, the submission is requested to identify, in a single table, a comprehensive list of additional evidence that could be valuable in informing reappraisals.

The table starts by identifying the key sources of uncertainty for effectiveness and cost-effectiveness. The methods guide determines that quantitative analyses should support the identification of the key uncertainties. Such analyses are well-understood and well-established in HTA (6). Uncertainty analysis requires that all sources of uncertainty are explicitly described (where possible) and included in the cost-effectiveness model to quantify the level of decision uncertainty (i.e., quantify the likelihood that the ICER is higher than specific values for the cost-effectiveness threshold), using probabilistic sensitivity analysis (PSA) and assessment of the robustness of the base case cost-effectiveness results in different scenarios. PSA is widely implemented in both centrally and locally developed cost-effectiveness models.

The guidance also requests that the expected consequences of overall uncertainty to the NHS are quantified using value of information analysis (7;8). All submissions should calculate the expected value of perfect information (EVPI). The EVPI is to be scaled up to reflect the population eligible for the treatment in the health system over a maximum of 10 years. To support the identification of relevant uncertainties, partial EVPI (EVPPI) could be used for key parameters (or groups of parameters) or, alternatively, univariate (one-way), best- or worst-case and multivariate (e.g., two-way) sensitivity analyses. Although preferred, the EVPPI is complex to calculate, and the methods guide therefore does not mandate its presentation. Where it is not possible to quantify uncertainty – for example, in the case of potential bias due to the use of a non/comparative clinical trial, that is, with a single arm study – this source of uncertainty should always be identified as relevant.

Besides identifying key sources of uncertainty, the table should also identify research designs that may mitigate against such uncertainties (which could include analyses of existing databases, the reporting of ongoing studies, or new data collection). Drawing from existing research (8), the new guide asks for submissions to consider future circumstances which may lead to changes in the clinical and/or economic value of the technology, and of research, for example, changes to the price of health technologies, new comparators emerging, or new evidence becoming available. In prioritizing future research, the new methods guide identifies the following considerations (8): the feasibility of the research (in particular, if the drug ends up being recommended for use in the NHS); whether there are significant irrecoverable costs incurred from introducing the technology in the NHS that may alter the cost-effectiveness in case the decision is reversed in the future; the possibility of changes in circumstances over time that could alter the value of the evidence (e.g., the introduction of generic medicines, new comparators, upstream changes in patient care, or relevant ongoing studies and research).

These aspects constitute a substantial departure from the 1998 guidance, which only required sensitivity analyses of key model parameters, and is novel in relation to most guidelines around the world (note, e.g., a comparable recommendation in the Dutch (9)).

### Presentation of evidence

The new methods guide specifically requests several summaries and analyses to best support decision-making.

To support contract negotiations, submissions are asked to identify the price at which the ICER of the new technology is equal to the cost-effectiveness threshold for the entire target population (higher volume and a lower price) and for when the decision is restricted to a cost-effective subgroup(s) (lower volume at a higher price). Because the threshold used of decision-making is not public, a range of thresholds between EUR 10,000 and EUR 100,000 per QALY gained is to be used. The price at which the EVPI is lower than the cost of conducting further research is also requested (unless the proposed price is already below it). Note that the level of decision uncertainty and the value of additional evidence are highest at prices equal to the cost-effectiveness threshold; therefore, lower prices will result in reduced uncertainty levels.

While for decision-making on NHS resources the perspective of the NHS is most relevant, the new guidance recognizes that it is important to acknowledge other potential costs and consequences of resource allocation decisions. Therefore, it allows, for clinical consequences, costs, and cost savings falling in other public and/or private sectors to be presented in scenario analyses (but not included in the ICER), disaggregated as follows: costs and/or savings for other sectors of the state; costs and/or savings for the patient, caregivers, and relatives (including patient co-payments or user charges); impact on the working capacity of the patient, caregivers and relatives, measured in days off work; and consequences for health and quality of life for caregivers and relatives.

The negotiation ultimately results in a contract that defines an NHS expenditure cap with that drug (the company rebates if the expense goes above the contracted value). Hence, the new guidance requires that BIA should always be included, limiting to a 2-year time horizon (to reflect the duration of the contract) and considering realistic scenarios on the rate of substitution of current clinical practice for current and prospective patients (determined based on prevalence and incidence, respectively). It must consider the costs related to the comparator(s) selected in the economic evaluation. The BIA considers the NHS perspective, but scenario analyses may separately consider impacts on other public sectors.

### Appropriateness of evidence to the Portuguese context

Typically, cost-effectiveness models are developed by manufacturers globally and adapted locally to the context of care of the jurisdiction. The new guidance explicitly considers how the adaptation to the Portuguese context is conducted to ensure that the evidence submitted best reflects the local context. It determines that submissions should explicitly attempt to identify evidence relevant to the Portuguese context; for example, by conducting specific searches of national journals complemented with consulting recognized experts to identify relevant published (or unpublished) literature as well as any relevant primary data sources. Where available, evidence from the Portuguese context should be compared to evidence from other contexts, with appropriate consideration for quality, quantity, and relevance.

The new guidelines make explicit consideration on the use of nonrandomized studies acknowledging that, for several reasons, there is an increasing number of technologies that have been granted marketing authorization based on such evidence. The guidance allows the use of evidence from nonrandomized studies to: (i) inform scenario analysis when nonrandomized trials are conducted in the Portuguese context, even in the presence of RCTs; (ii) inform the reference case when there is no evidence produced in RCTs, accompanied by a sensitivity analysis that varies the effect parameter up to the value corresponding to the assumption that the new technology has no effect; (iii) inform the time extrapolation of the treatment effect beyond the follow-up period of RCTs, complemented by a sensitivity analysis to alternative assumptions about the duration of the treatment effect; and (iv) inform disconnected networks of evidence. (10;11)

When no empirical evidence on a parameter of interest exists, or its representativeness to the Portuguese context is uncertain, the opinions of experts can be elicited. There is a long-standing tradition of using expert panels in Portugal to inform submissions (particularly focusing on resource use), but no guidance on how to implement these exists. The new guidelines specify that a structured and explicit process should be used. (12) Experts should be asked to express their judgements quantitatively and uncertainty in knowledge should be elicited (to help decision-makers take stock of the level of uncertainty in the decision). The implications of between-expert variation are to be assessed using scenario analyses. To ensure consistency across appraisals, the guidelines specify a set of reference methods (see the full guideline document in (3)), but recognizing little is known on the accuracy of alternative elicitation methods, scenarios using alternatives elicitation methods are welcomed, e.g., calibration methods (13).

When the evidence base consists of RCTs but forms two or more disconnected elements (that is disconnected network, see above and (10;11)), the guidelines recommend, by order of preference: 1. broadening the evidence network to consider 2^nd^ and 3^rd^ order indirectness; 2. broadening the evidence base to include, for example, evidence from other populations (e.g., borrow evidence from an adult population adult to inform treatment effects in children); 3. introduce assumptions regarding treatment effects (e.g., equivalence or exchangeability); or 4. direct use of ‘unanchored’ adjustment methods, use of observational data or elicited expert opinion. Full justification is required when using these approaches and results should be interpreted carefully.

The adequacy of evidence on health-related quality of life (HRQoL) weights was also addressed in the guidance by determining that the preferred measure is the EQ-5D-5L (14), for which there is a published Portuguese tariff (15). If this is not available, other generic preference-based measures such as SF-6D (16) or HUI (HUI 1, HUI 2 or HUI 3) (17–19) are preferred to mapping to the EQ-5D-5L from other instruments.

### Transparency and validity

Transparency in reporting and appropriate validation procedures were another concern taken into consideration when devising the new guidelines.

The guidance sets out that the choice of modelling approach should always be justified and presented alongside a full description of how the model reflects the natural course of the disease, the impact of treatment(s) on the disease, health outcomes and health costs; as well as a full description of the sources of information, assumptions made (with their rationale) and a list of model parameters (highlighting all treatment-related parameters). The guidance recognizes that an important role of modelling is that it permits extrapolation of costs and health consequences over a longer time horizon. Assumptions and evidence used to support extrapolations is to be identified explicitly in the submission. Where partitioned survival models are used, the plausibility of its extrapolations should be judged and carefully justified.

Statistical analyses performed on individual patient level data that have not been fully published in peer-reviewed literature should be fully documented in a statistical appendix. The design, conduct and results of expert elicitation need to be reported in detail, that is the protocol, a summary of the conduct of the elicitation process (e.g., who facilitated the exercise, any deviations to protocol accompanied by justification, etc.) and results of the exercise should be clearly reported.

Data relevant to the Portuguese context should be explicitly sought and, where available, incorporated in analyses (considering of their quality). Given the likely use of international data, the face-validity of input data and model results should be assessed in relation to the Portuguese context by comparing the model results with external empirical data (e.g., observational data), or expert opinion when empirical data are not available.

Model validation is crucial to demonstrate that the model results are reliable, credible, and transferable to the Portuguese context. The guidance sets out that validation should be conducted on all elements of model development, including the conceptual model, selection of input data, electronic model implementation, and model outcomes. If the implementation of a model has been validated as part of the assessment by other regulatory authorities, this can be omitted with reference to earlier validation processes. However, model validations should always highlight the transferability and generalizability of model predictions to the Portuguese context, and the validation of possible model adaptation to Portugal (specific parameters and hypotheses). For that, the judgements of experts may also be used.

## Discussion

Public consultation on the new Portuguese guidelines was performed with key stakeholders (between May and July 2019); we here present a selection of those comments judged to be most significant. As a response to the consultation, modifications to the methods guide were made by the Ministry of Health. Below, we describe the changes made, but we are not able to justify these as this information was not made public.

### Challenges of considering multiple comparators

The draft guidance document that was out for public consultation requested full incremental analyses with multiple comparators. Stakeholders argued against this, concerned with increased complexity and uncertainty, and suggested a single comparator, either the drug most frequently used in clinical practice for the indication or the drug which is most likely to be replaced by the new drug under evaluation. While replacing current practice may *appear* cost effective, pairwise analysis will not highlight the possibility of current practice being itself inefficient. The inclusion of all comparators allows a fuller discussion about how to improve the systems’ efficiency and contribute to transparency in decision-making. The final draft of the new guidance specifies that efficiency needs to be used to define the relevant comparator, and if this can be determined *a priori* then a single comparator is allowed.

### Changing from a societal to an NHS perspective

In contrast with the old guidelines that considered a societal perspective, the new guidelines adopt an NHS perspective. Stakeholders argued that the responsibilities of NHS should not be restricted to the management of the NHS budget but aim to optimise society’s resources. The new guidelines argue that, while recognizing that NHS decisions may have spillover effects to other sectors in society, departures from the healthcare payer perspective poses political, methodological, and empirical challenges. These arise from not just the need to formalize (or prescribe) trade-offs between health, consumption, and other social arguments, which may generate conflicts between social objectives, but also from measurement challenges associated with the adoption of a societal perspective which increases the lack of transparency and comparability between submissions (20).

However, recognizing the possibility of spillover effects, the new Portuguese guidelines ask that any impacts on other sectors (e.g., patient costs, changes in ability to work) are made known to the decision-maker. Where justifiable, the company can present evidence on these but needs to do so separately and not aggregate these into an alternative ICER. One key concern on this topic related to the existing co-payment system. Under an NHS perspective, drugs with higher patient co-payments will need to demonstrate lower health benefits than those requiring higher levels of NHS investment. Given that the new guidelines make explicit these patient costs, decision-makers can have appropriate consideration for drugs with higher levels of patient co-payment that show low magnitude of health gains.

### Discount rates

The draft version of the new guidelines specified a discount rate of 5percent. Stakeholders challenged this value as being too high and suggested that the discount rate should be lower for benefits that for costs. The final guidance adopted a common value of 4 percent for costs and outcomes, used by the Government for the Private Partnerships and by the EU for Investment Funds (Decree 480/2014 of March 3, 2014).

### Defining a preferred instrument for HRQoL and a national valuation algorithm

While the old guidance was not prescriptive about the instrument used to quantify HRQoL weights, the new guidelines prefer the EQ-5D. Stakeholders argued that defining a preferred generic preference-based measure may impose restrictions in cost-effectiveness studies, especially when this measure is not considered the most relevant to the population. However, it will ensure consistency across appraisals. The use of national tariffs allows is better reflective of the context of care.

### Burden of the evidence requirements in the new guidelines

Stakeholders claim that the guideline proposal significantly increases the scrutiny of models (that are typically developed internationally, with often limited scope for modifications) and requests excessively burdensome information and analyses. However, the evidence requests defined in the new guidelines had explicit consideration for the constraints of centrally developed models, with additional requirements (e.g., to present EVPI or sensitivity analysis to price) being easily obtained from these models. Formal model validation is required, but internal validation is noncompulsory if the model has been scrutinized by other agencies. Other additional requirements, such as seeking evidence pertaining to the Portuguese context of care, the formalization of expert elicitation exercises and the additional exploration of uncertainty and prioritization of evidence requirements, are essential to decision-making.

### Conclusions

Twenty years after the publication of the first, and pioneering, Portuguese methods guidance for economic evaluation of health technologies, a revision was deemed warranted. The revised methods guidance aimed at introducing novel methods and modelling techniques, at informing reappraisal procedures, at accounting for new evidence specific to the Portuguese context, and at providing further considerations over health system budget constraints under limited resources, among other aspects. The new methods guidance adopted innovative techniques, with as yet little adoption by other HTA agencies across Europe (e.g., regarding parameter and structural uncertainty to support reappraisals). The development of the new methods guidance involved the participation of multiple stakeholders and was shaped by their feedback. The highly participatory approach allowed, for example, considerations over the analysis perspective, the use of multiple comparators and the discount rate for costs and benefits.

The new methods guide was implemented in Portugal in 2020. To facilitate its implementation, the guide was presented to the HTA Committee (CATS) and technical elements were discussed with health economists. Although no other implementation activities were planned, opportunities exist within the process for stakeholders and health economists conducting appraisals for CATS and companies to raise questions about the application of the methods guide. Up to now, no major issues have been raised. No further evidence on implementation has been purposefully collected.
